# Role of Nutrition in the Management of Chronic Liver Disease

**DOI:** 10.1016/j.gastha.2024.100613

**Published:** 2025-01-02

**Authors:** Matt Pelton, Sarah Abdel-Meguid, Eshani Goradia, Arvind Bussetty, Deborah Cohen, Keerthana Kesavarapu

**Affiliations:** 1 Department of Internal Medicine, Rutgers Robert Wood Johnson, New Brunswick, New Jersey; 2 Department of Clinical and Preventive Nutritional Sciences, Rutgers School of Health Professions, Newark, New Jersey; 3 Department of Gastroenterology and Hepatology, Rutgers Robert Wood Johnson, New Brunswick, New Jersey

**Keywords:** Cirrhosis, Malnutrition, Sarcopenia, Protein, Calories

## Abstract

Malnutrition is prevalent, detrimental, and associated with worse outcomes, including higher rates of hospitalization, morbidity, and mortality. In this review article, we aimed to define malnutrition in patients with chronic liver disease (CLD), elucidate the pathogenesis of malnutrition, discuss the advantages and disadvantages of current screening methods, and highlight the latest evidence-based dietary recommendations. Emerging evidence suggests that CLD-specific tools such as the Liver Disease Undernutrition Screening Tool and the Royal Free Hospital-Nutritional Prioritizing Tool can accurately identify patients at high risk for malnutrition and should be used in conjunction with more standard tools such as subjective global assessments. The pathogenesis of malnutrition in CLD is multifactorial but seems to arise in large from altered metabolism, namely a reduction in protein synthesis and an increase in resting energy expenditure. However, decreased nutrient intake, impaired nutrient absorption and increased nutrient losses have also been shown to contribute. Key findings in this review argue against protein-restricted diets in patients with CLD and support the use of plant-based proteins over dairy and meat proteins for those with liver cirrhosis complicated by hepatic encephalopathy. Frequent small meals are recommended in patients with liver cirrhosis in addition to the avoidance of prolonged fasts >12 hours due to their hypercatabolic state. CLD covers a wide spectrum of diseases, and this review calls for an individualized approach to addressing the specific nutritional needs, depending on the etiology of CLD, its severity, associated complications, and comorbid conditions. This can be best achieved by close, longitudinal follow-up with a multidisciplinary team including a registered dietitian who can obtain a comprehensive, accurate nutritional assessment.

## Introduction

Malnutrition affects a substantial proportion of people with chronic liver disease (CLD), with a prevalence ranging from 13.3% to 85% (average 37.6%). As CLD progresses to liver cirrhosis (LC), malnutrition rates increase to a range of 26.7%–93.6% (average 44.1%).^1^ These wide ranges of estimates of prevalence are partly due to many methods for testing for malnutrition and different operational definitions. Sarcopenia—the progressive loss of skeletal muscle mass and strength—is closely linked to malnutrition and highly prevalent among patients with CLD and LC.^2^

There is growing interest in the impact of nutritional abnormalities on CLD severity. Both malnutrition and sarcopenia are associated with higher rates of complications such as susceptibility to infections,^3^ hepatic encephalopathy (HE),^4^ and ascites.^5^ Furthermore, they are independent predictors of lower survival in LC^6^ and among those undergoing liver transplantation.^7^ While malnutrition and sarcopenia influence the prognosis and management of CLD, the role of targeted nutritional management in improving patient quality of life (QoL) and decreasing complications of LC is unclearly described. This review aims to describe the impact of malnutrition, sarcopenia, and frailty on persons with CLD and LC, and outline the evidence supporting multidisciplinary management of nutrition status.

## Defining Malnutrition, Sarcopenia, and Frailty

Operational definitions of malnutrition, sarcopenia, and frailty differ among professional organization guidelines and individual practice. This ambiguity is in part what makes screening for these constructs particularly challenging in this patient population, contributing to wide ranges of reported prevalence of malnutrition in CLD. Here, we adopt the definition of malnutrition deployed by the American Society of Enteral and Parenteral Nutrition, which includes both overnutrition and undernutrition under the umbrella term of malnutrition (Table 1).^8^ For this review, sarcopenia is defined as a progressive reduction in muscle mass as evidenced by computed tomographic (CT) imaging of the third lumbar vertebrae. Frailty in patients with LC is defined as impaired physical function due to muscle weakness and generalized muscle loss, frequently assessed using the Liver Frailty Index.^10^

**Table 1:** Definition of Malnutrition, Sarcopenia and Frailty in Patients With CLD

Construct	Definition in patients with CLD
Malnutrition	• “An acute, subacute or chronic state of nutrition in which a combination of varying degrees of overnutrition or undernutrition with or without inflammatory activity have led to a change in body composition and diminished function”^8^^a^
Sarcopenia	• Progressive reduction in muscle mass and subsequent function as found on CT imaging of the third lumbar vertebraesex-specific, skeletal muscle index cut-offs <50 cm^2^/m^2^ for men and <39 cm^2^/m^2^ for women.^9^
Frailty	• Clinically recognizable state of limited or impaired physical function due to decreased physiologic reserve

a Adapted From Soeters PB, Schols AM. Advances in Understanding and Assessing Malnutrition.

It is recommended that all patients with CLD, regardless of etiology, be screened for malnutrition and undergo a comprehensive nutrition assessment. It is also recommended to assess frailty and monitor these patients for signs of sarcopenia to identify potential risk of malnutrition. Nutrition is especially vital for people undergoing liver transplantation, but this will not be the focus of this discussion.

## Screening for Malnutrition

### Body Mass Index (BMI) and Child-Pugh Classification

Per the European Association for the Study of Liver Diseases’(EASL) 2019 clinical practice guidelines, body mass index (BMI) and Child-Pugh Group (CPG) classification are recommended as frontline risk stratification tools to identify patients with CLD at higher risk for malnutrition and sarcopenia. While BMI gives a rough proportional estimate, it is important to note that the presence of ascites can increase body weight without providing functional capacity, and therefore BMI alone may falsely underestimate the risk of sarcopenia and frailty. Some researchers attempt to correct actual body weight for the severity of ascites, though this approach is not standardized, and it is unclear the impact of this on outcomes.^11^

It is generally understood that any patient with a BMI less than 18.5 kg/m^2^ or with CPG-C decompensated LC, is classified as the highest risk for malnutrition.^12^ However, this does not mean that those falling outside these ranges are not at risk. One observational study found that 90% of patients with LC with a BMI <18.5 kg/m^2^, 49.1% with a BMI of 18.5–25 kg/m^2^, and 40.6% with a BMI >25 had some evidence of malnutrition.^13^ Another systematic review found that the mean prevalence of sarcopenia in patients with varying stages of LC was anywhere from 33% to 46% depending on the severity of the disease.^14^ These findings suggest a significant yet wide range of prevalence of malnutrition and sarcopenia in patients with CLD. For this reason, further screening methods are required to accurately detect these constructs.

There has been an advent of screening tools to stratify patients with CLD and LC at risk for malnutrition; however, most of these scoring tools have not been adequately validated in this patient population, chiefly because they either rely on subjective assessments or weight which is frequently impacted by fluid retention and hypoalbuminemia.

### Subjective Global Assessments

The Subjective Global Assessment (SGA) questionnaire is a widely used nutritional assessment tool to identify patients with malnutrition regardless of the etiology. It categorizes patients as well-nourished (SGA-A), mildly to moderately malnourished (SGA-B), or severely malnourished (SGA-C).^15^ It is easy to complete and has been shown to be a reliable tool for predicting morbidity and mortality associated with malnutrition.^15^^,^^16^ However, some studies have found that the SGA only weakly correlates with sarcopenia in patients specifically with CLD and that this relationship was nonsignificant in obese patients with CLD.^17^

Due to the limitations of the SGA for patients with CLD, the Royal Free Hospital-SGA (RFH-SGA) was created, which similarly stratifies patients into 3 groups. The notable difference between the SGA and the RFH-SGA is that the latter stratifies patients based on dry weight BMI and mid-arm muscle circumference (MAMC). A recent study conducted on inpatients with CLD found that the RFH-SGA but not the SGA was a good predictor of sarcopenia.^16^ As such, this tool is a simple bedside screening tool to identify malnutrition in patients with CLD, but further studies are needed for validation, specifically in the outpatient setting.

### RFH-Nutritional Prioritizing Tool

The RFH-Nutritional Prioritizing Tool (RFH-NPT) is a promising CLD-specific screening tool. It categorizes patients into low (0 points), medium (1 point), or high risk (2–7 points) for malnutrition. It takes the typical variables including weight loss, decreased nutritional intake, and BMI into account but also considers whether the patient has acute alcoholic hepatitis, started tube feeds, has evidence of volume overload, or has any recent history of acute illness.^18^ It is quick, easy, and does not need to be performed by a specialist. Recent evidence suggests that in the inpatient setting, the RFH-NPT correlates well with the severity of CLD, CPG class, and Model for End-Stage Liver Disease scores.^19^ Furthermore, there was evidence to suggest that improvement in RFH-NPT scoring led to improved survival.^19^ Although the RFH-NPT seems to be promising in the inpatient setting, more observational studies are needed to validate its utility in patients with CLD in the outpatient setting.

### Liver Disease Under Nutrition Screening Tool (LDUST)

The Liver Disease Under Nutrition Screening Tool (LDUST) is a self-administered CLD-specific tool that uses 6 patient-directed questions to assess nutritional status. These questions specifically focus on patient-reported outcomes including intake, weight loss, fat loss, loss in muscle, abdominal or leg swelling, and ability to carry out activities of daily living.^20^ Responses are categorized as columns A, B, or C with 2 or more B/C answers indicating undernutrition risk. One prospective cohort study demonstrated evidence that the LDUST can predict outcomes of patients with LC including death, progressive decompensation, and hospital readmission.^21^ Another study found that the LDUST was accurate in detecting the risk of malnutrition in patients with CLD in the outpatient setting.^22^ A major drawback to this screening method is that it relies entirely on the patient’s subjective assessment and so has low inter-rater reliability.

## Screening for Sarcopenia

### CT Lumbar Spine

Malnutrition and sarcopenia coexist in CLD. Table 2 summarizes the various modalities of assessing sarcopenia and their strengths and limitations. CT imaging at the third lumbar vertebra to assess psoas muscle and abdominal wall muscle loss is considered the gold standard. Using imaging, the skeletal muscle index is calculated, which normalizes muscle area to patient height. Sex-specific skeletal muscle index cut-offs in patients with LC are only weakly validated and it was recently suggested to be 50 cm^2^/m^2^ for men and 39 cm^2^/m^2^ for women in 1 study.^22^ Limitations of using CT to identify sarcopenia include access and its high cost.

**Table 2:** Advantages and Limitations of Methods Used to Screen for Malnutrition and Sarcopenia in Patients With CLD

Screening methods	Condition intended to identify	Advantages	Limitations	Specific population considerations
BMI	Malnutrition and Sarcopenia	A simple, inexpensive, noninvasive way to assess whether patients are underweight, healthy weight, or overweight	Does not account for fluid retention (ie ascites, leg edema)	Has not been validated in patients with CLD
CPG	Malnutrition	CLD-specific, largely based on objective/lab values	Designed to predict mortality in patients with LC not nutritional status, has some subjective components	N/A
SGA	Malnutrition	Reliable tool to predict morbidity and mortality	Weak correlation with sarcopenia in CLD	General population but not well correlated in CLD patients
RFH-NPT	Malnutrition	CLD-specific, quick, noninvasive, accounts for fluid retention	Includes some subjective components	Weakly validated in patients predominantly with alcohol-related liver disease.^23^ Has not been validated in other populations
Liver Disease Under Nutrition Screening Tool	Malnutrition	CLD-specific, quick, noninvasive, does not require a specialist	Relies entirely on subjective information provided by the patient	Weakly validated in Caucasian patients with LC in the outpatient setting^23^
CT scan at L3 to assess sarcopenia	Sarcopenia	Accurate, quick, measures muscle quantity and quality	High radiation exposure, expensive, varying cut-points/sites of measurement, not easily repeatable, not portable	N/A
DEXA	Sarcopenia	Safe (low radiation), quick	Edema can limit accuracy, cannot be performed at bedside/not portable, expensive	N/A
MAMA, MAMC, TSF	Sarcopenia	Relatively simple to perform at bedside or in outpatient setting, low cost, not impacted by edema	Must be performed by trained individuals otherwise poor intrarater and inter-rater reliability	N/A

MAMA, mid-arm muscle area.

### Dual-Energy X-Ray Absorptiometry (DEXA) Scan

Another way to assess sarcopenia is via whole-body dual-energy x-ray absorptiometry (DEXA), which provides an analysis of body composition and quantifies fat mass, lean mass, and bone mineral content. With this imaging modality, any nonbone or nonfat matter is assessed as lean mass. For this reason, ascites and edema in patients with CLD may limit accuracy.

### Anthropometric Methods

Unlike CT and DEXA imaging, body mass assessment can be done at the bedside via anthropometric methods most used measurements include the MAMC, the mid-arm muscle area, and triceps skinfold thickness (TSF). The MAMC = mid-arm circumference - [TSF × 0.314] while mid-arm muscle area = MAMC^2^ ÷ [4 × 0.314].^24^ There is some evidence that MAMC and TSF correlate with survival in patients with LC though MAMC was deemed to have higher prognostic power in 1 study.^25^ Additionally, other studies have shown that TSF and MAMC measurements in patients with LC decrease with increasing severity of LC according to CPG.^26^ The advantages to using body measurements to screen for sarcopenia are that it is portable, can be done at the bedside in the ambulatory care setting, and is relatively inexpensive. These measurements should be taken by professionals trained in skinfold assessment for good intrarater and inter-rater reliability. A major drawback to this approach is that there are no established cutoff values for TSF and MAMC in patients with CLD.^27^ For this reason, a combination of methods may be employed to assess nutritional status and sarcopenia.

## Sarcopenic Obesity (SO)

Sarcopenic obesity (SO), which occurs in the setting of decreased muscle mass alongside increased adiposity, is closely linked to insulin resistance and metabolic syndrome.^28^ Furthermore, growing evidence suggests an association of SO with the development and progression of metabolic dysfunction–associated steatotic liver disease (MASLD).^28^ It is therefore important to highlight that overweight and obese patients with CLD can still have malnutrition. This is another reason a thorough assessment of sarcopenia and SO via these modalities may be valuable in the management of patients with CLD.

## Summary of Screening Modalities

Detecting malnutrition and sarcopenia in patients with CLD is a challenging task. The best approach involves using a combination of tools including global assessment, imaging, body composition assessment methods, as well as one of the CLD-specific tools. Although further validation is needed, both the RFH-NPT and the LDUST have been shown to accurately identify malnutrition in patients with LC with a high sensitivity, in the inpatient and outpatient settings respectively.^20^ To devise targeted detailed nutrition care for patients with CLD at risk of malnutrition, it is important to review the pathophysiology contributing to these deficits.

Each assessment tool has unique advantages and disadvantages, and the variety of methods allow for use in many clinical settings. In the outpatient setting to assess nutritional status, SGA is the most reliable tool as it is not limited to etiology and has a strong predictor capability for morbidity and mortality associated with malnutrition. The tool is easy to perform but limited in CLD patients due to a weak correlation to sarcopenia. The modified RFH-SGA can account for this limitation to be used particularly in CLD patients.

## Pathophysiology of Malnutrition

Nutritional management of the patient with LC demands an in-depth understanding of its metabolic challenges, primarily due to the condition’s accelerated starvation state. This state triggers a metabolic shift from glucose to fatty acids, a reduction in protein synthesis and an increase of gluconeogenesis from amino acids, leading to sarcopenia and elevate resting energy expenditure (REE).^29^^,^^30^ The condition is further aggravated by factors such as diminished dietary intake due to dysgeusia, anorexia, nausea, dietary restrictions along with complications such as portal hypertension, esophageal varices, medication side effects, decreased nutrient absorption, and alterations in the gut microbiome.^31^

### Energy Intake

Patients with LC experience a unique metabolic scenario characterized by hypermetabolism, complicating the balance between total energy expenditure—which includes REE, food-related thermogenesis, and energy from physical activity—and energy supply, typically necessitating between 28 and 37.5 kcal/kg.BW/d.^23^^,^^32^, ^33^, ^34^, ^35^ Approximately 15%–30% of persons with LC exhibit hypermetabolism, where their REE exceeds 120% of predicted values, significantly impacting their nutritional status and contributing to malnutrition and weight loss (specifically lean body mass).^33^^,^^36^, ^37^, ^38^

The precise mechanisms driving hypermetabolism in LC remain partially understood. However, inflammation is a suspected catalyst.^39^ LC, particularly when secondary to alcohol-associated liver disease (ALD), often involves elevated levels of proinflammatory cytokines which contribute to malnutrition not only by increasing metabolic demands but also by reducing appetite.^40^, ^41^, ^42^ The intricate relationship between LC, inflammation, and the gut–liver axis is incompletely understood and requires further research to fully characterize the pathophysiology and identify potential therapeutic strategies.^43^, ^44^, ^45^

### Protein

The pathophysiology surrounding protein metabolism in patients with LC involves complex interactions between protein loss, altered metabolism, and hormonal changes. Portal hypertensive enteropathy contributes to protein loss, exacerbating the challenges of maintaining adequate protein levels.^46^, ^47^, ^48^ LC is marked by increased protein catabolism alongside diminished protein synthesis, further complicated by imbalances in amino acids. Specifically, decreased concentrations of serum branched-chain amino acids (BCAA) and elevated levels of aromatic amino acids (AAAs) are notable, contributing to the development of HE and muscle wasting.^49^, ^50^, ^51^, ^52^ Additionally, testosterone, which contributes to protein synthesis and breakdown, is significantly reduced in approximately 90% of men with LC, impacting lean body mass maintenance.^53^^,^^54^

Leading health organizations in the United States and Europe, including the European Society for Clinical Nutrition and Metabolism (ESPEN), the American Association for the Study of Liver Diseases, the International Society for Hepatic Encephalopathy, and EASL, have developed comprehensive guidelines for the nutritional management of LC. ESPEN specifically recommends a protein intake of 1.2 gm/kg for individuals with compensated cirrhosis and increases this recommendation to 1.5 gm/kg for those who are malnourished and/or sarcopenic. Guidelines also advise against restricting protein in patients with HE due to the risk of exacerbating protein catabolism. Critically, ESPEN provides detailed guidance on assessing nutritional needs, recommending the use of actual body weight for those without ascites and ideal body weight for those with ascites to ensure accurate nutrient needs assessments.^55^^,^^56^ For patients with CLD and ascites, the clinician can use the patient's dry weight or adjust actual weight by subtracting 5% for mild, 10% for moderate, and 15% for severe ascites.^55^ This ensures adequate dietary intake, particularly for protein, that align with the patient's actual physiological needs.^56^

The approach to protein intake in LC, especially regarding protein restriction, has evolved significantly. Historically, protein restriction was commonly recommended for those with LC, particularly those with HE.^57^^,^^58^ However, recent studies have shown that malnourished patients with LC are more susceptible to HE, with a notable inverse relationship between muscle mass and blood ammonia levels.^59^ In a landmark study, 30 patients with LC hospitalized with overt HE (OHE) were randomized to receive either a low-protein or a normal-protein diet over 14 days, alongside standard treatments for OHE.^60^ Protein metabolism, assessed via the glycine-N (15) infusion method, revealed no significant difference in HE outcomes between the 2 groups. However, the low-protein diet group experienced increased protein breakdown, indicating that a protein intake at or above the daily recommended intake level is both safe and metabolically preferable for patients with OHE. This study effectively demonstrated that protein restriction does not improve HE but may worsen overall metabolic health by further exacerbating protein catabolism.^60^

### BCAA Supplementation

In managing LC and addressing malnutrition, BCAA supplementation is a controversial topic. Patients with LC frequently encounter a nutritional imbalance characterized by high levels of AAAs—phenylalanine, tyrosine, and methionine—and reduced levels of BCAAs, affecting neuronal excitability and metabolic health.^61^ Unlike AAAs, the BCAAs such as valine, leucine, and isoleucine are metabolized by skeletal muscle, bypassing liver metabolism.^62^ Despite the theoretical promise that BCAA supplementation seems to provide regarding balancing amino acid levels, literature calls for more rigorous studies to refine BCAA usage guidelines.^61^ Gluud et al. conducted a study administering BCAAs at a dose of 0.25 g/kg body weight/day and compared it with placebo or no intervention. The findings indicated an improvement in recurrent HE, particularly in OHE compared to minimal HE (MHE), without a significant impact on survival rates.^63^ Another more recent study by Siramolpiwat et al. revealed that frail, compensated patients with LC experienced an enhancement in frailty following 16 weeks of BCAA supplementation. Furthermore, this regimen led to gains in muscle mass and improvements in the physical aspect of QoL for these individuals.

The disparity in the current literature underscores the urgent need for more comprehensive studies to establish definitive guidelines for BCAA supplementation in the nutritional management of LC [63]. Present guidelines recommend BCAA supplementation in instances of decompensated LC or inadequate dietary protein intake, with a preference for proteins rich in BCAAs from vegetarian sources for better management of HE due to improved tolerance. In practice, BCAAs may be incorporated into shakes or oral nutritional supplement beverages. However, consumption of BCAAs is not a common practice, largely because BCAAs are known for their unpleasant taste and high cost, resulting in poor compliance and in addition, BCAAs are typically not available on many acute care hospital formularies. Further research in controlled settings is needed to determine if the benefits of BCAA supplementation are cost-conscious and feasible in clinical settings.

### Plant, Dairy, and Meat Proteins

In the dietary management of LC, the protein source in the diet may influence clinical outcomes, particularly for patients with HE. EASL provides a weak recommendation to consider dietary modification toward vegetable proteins in refractory cases of HE because of 2 small studies favoring vegetable proteins over dairy and meat proteins for those with HE.^31^^,^^64^^,^^65^ It is, however, important to note the 2 crossover studies were relatively small (8 and 10 patients) and that the treatment of HE has advanced since they were published in 1993 and 1982. If using this approach to treat HE, it is important to ensure it is well-tolerated and that patients maintain adequate caloric and protein intake via vegetable sources. The field would benefit from validation of these findings in modern populations to support a stronger recommendation favoring vegetable protein sources and investigate the feasibility of implementing them in clinical practice.

## Micronutrients

### Vitamins

Vitamin deficiencies in individuals with CLD stem from compromised liver function as the liver is intimately involved in micronutrient metabolism but also secondary to reduced nutrient storage, absorption as well as insufficient intake. For example, deficiencies in vitamin A and D have been reported in 69.8% and 81.0% of liver transplant candidates, respectively.^66^ However, it is unclear if supplementation of vitamin D impacts treatment responses, and further work is needed to evaluate causation of the vitamin D deficiency.^67^ EASL advises checking vitamin D levels in patients with MASLD, ALD, and cholestatic disorders. Oral supplementation is recommended in patients with serum vitamin D (25(OH)D) levels below 20 ng/mL, targeting levels above 30 ng/mL.^31^

Historically, the increased international normalized ratio (INR) seen in patients with CLD has been attributed to dietary deficiencies and inadequate levels of vitamin K. However, several studies have found that less than 15% of patients with LC have a vitamin K deficiency.^68^ Evidence suggests that liver dysfunction and isolated LC are not automatically associated with increased risk.^69^ A review by Ng examines the development and the misguided adoption of the “1.5×” benchmark for INR as a criterion for administering fresh frozen plasma/frozen plasma, suggesting that this method is outdated and not supported by new literature. Routine supplementation of vitamin K1 or phytonadione administration to normalize INR in patients with LC not on warfarin is not generally recommended due to lack of evidence suggesting benefit. Furthermore, oral phytonadione has literature suggesting it is generally ineffective in patients with LC due to poor absorption and is typically not recommended.^70^

### Timing of Energy Intake

Timing of energy intake may impact liver function, particularly in patients with metabolic dysfunction–associated steatohepatitis (MASH) and MASLD. Initial approaches in patients with MASLD/MASH, should target weight reduction and promote a dietary pattern that decreases insulin resistance.^71^ In this regard, there is evidence that regular distribution of food throughout the day is inversely associated with insulin resistance. Furthermore, irregular eating patterns, including skipping breakfast and late-night snacking that go against circadian rhythms and physiologic daily functions may contribute to CLD.^72^ Thus, patients with early MASH/MASLD-related CLD may benefit from regular meal patterns.

However, in patients with progression of any CLD to LC, increased LC-related catabolism may necessitate more frequent meals than the standard 3 daily meals. Specifically, the consensus put forward by ESPEN recommends that patients with LC should not fast for more than 12 hours. This is due to the hypercatabolic state of LC in which protein stores are broken down at accelerated rates for gluconeogenesis. Several studies have explored the benefit of interval eating to avoid fasting periods, as well as late-night eating. One randomized controlled trial compared late-night supplementary nutrition using 2 cans of Ensure Plus with daytime supplementation, measuring change in total body protein (TBP) over 12 months. Daytime nutrition supplementation did not significantly change TBP over 12 months compared to baseline levels, while nighttime supplementation resulting in a 2 kg gain in lean tissue over a 12-month period compared to the baseline levels. Thus, nutritional supplementation at night may be associated with a greater TBP composition and reduce the protein-energy malnutrition barrier.^73^

A recent meta-analysis found laboratory and clinical benefits of late-evening snacking in patients with LC.^74^ Late-evening snacking was defined as food/energy intake typically between the hours of 9:00–10:00 PM. Across 8 studies with a total of 341 patients, late-evening snacking increased albumin, while also reducing ammonia, prothrombin time, and aminotransferases. More importantly, they found late-evening snacking had a lower chance of ascites and HE occurring. Further work and longer follow-up is required to evaluate the duration of these improvements, and their effect on clinical outcomes.^75^

## Individualized Approach to Nutrition in Patients with LC

Optimizing nutritional approaches in patients with CLD and LC requires an individualized approach based on a patient’s etiology of CLD, complications of CLD and comorbidities—which may uniquely impact their nutritional status. The underlying etiology or contributing etiologies of a patient’s disease must be determined and nutritional status assessed to provide individualized recommendations.

## Etiology

### ALD

For patients with ALD, special dietary considerations should include vitamin supplementation and careful assessment for sarcopenia. Due to a combination of alcohol-mediated enteropathy and reduced oral intake, patients with ALD have a higher frequency of deficiencies of vitamins B12, folic acid, and thiamine. Thus, providers should consider ensuring adequate levels with testing (particularly if patients have macrocytosis) and/or empiric supplementation. Secondly, patients with ALD may be at higher risk of sarcopenia; studies have found a 47%–80% prevalence of sarcopenia in ALD compared with a 22%–60% prevalence in patients with LC secondary to chronic hepatitis B, chronic hepatitis C, MASLD, or autoimmune hepatitis (Table 3).^76^^,^^77^^,^^79^ This is thought to be in part due to extensive alcohol-related skeletal myopathy, which can be partially reversed with a sustained response by complete abstinence or a substantial reduction in the amount of consumption.^78^ Patients with ALD should be carefully monitored and treated for vitamin deficiencies in addition to vigilant assessment for sarcopenia.

**Table 3:** Considerations for Personalizing Approaches to Patients With CLD/LC Based on Etiology, Severity, and Complications of Disease, as Well as Comorbid Conditions

Etiology	Sarcopenia	Frailty	Special considerations
Alcohol	Highest prevalence^76^ Partially Reversible^77^^,^^78^	Limited evidence	Vitamin B1, B9 and B12 supplementation and careful assessment of sarcopenia
Metabolic	Lower prevalence (22%–60% vs 47%–80% in ALD)^79^	Higher risk (49% vs 34% ALD)^79^ and higher risk of worsening over time^80^	Higher prevalence of SO (aOR 6.0, 95% CI 1.4–25.3)^81^ and may benefit from aggressive lifestyle interventions
Hepatitis B/C	Lowest prevalence (7.1%–10% without LC, 11.8%–17.7% compensated LC, 21.9% decompensated LC)^82^^,^^83^	Limited evidence	Less well studied in high income countries
Severity			
Child-Pugh Class	A: 28.3%–33% B: 36%–37.9% C: 46%–46.7%^14^^,^^84^	Limited data	Consider early screening, and more urgently screening as disease progresses
Complications			
Hepatic encephalopathy	Likely higher prevalence (OHE 30% vs 15% MHE 49% vs 30%)^4^^,^^85^	Increased risk (aOR 2.45 95% 1.80–3.33)^86^	Evidence for BCAA supplementation Aspiration precautions Early parenteral nutrition supplementation if required Avoid protein restriction
Ascites	Likely increased risk (aOR 8.11 95% 1.7–51.9)^85^^,^^87^	Increased risk (aOR 1.56 95% 1.15–2.14)^86^	Diuretics and large volume paracentesis to reduce abdominal discomfort, improve exercise tolerance Consider risk/benefit of salt restriction. For protein supplementation, use dry weight or subtract weight by 5% for mild, 10% for moderate and 15% for severe ascites
Hepatocellular Carcinoma	Higher prevalence (38% vs 33%)^14^	Limited evidence	Especially in patients that are not candidates for transplant, encourage early liberalization of dietary restrictions
Demographics/Comorbidities			
Gender	Men: 41.9%–61.6% Women: 23.6%–36%^14^^,^^88^	Males at increased risk of frailty^89^	Individualized management
Obesity	Mixed evidence showing both increased and reduced risk^81^^,^^85^	Limited evidence	Individualized management

### MASLD and Multifactorial Etiologies

Recent changes in the classification of CLD resulting from multiple comorbidities have been suggested. According to the updated multisociety Delphi consensus statement, all patients with CLD and a BMI >25 should be considered as having a component of MASLD contributing to their hepatosteatosis; regardless of other comorbid etiologies of CLD. This change highlights the multifactorial nature of CLD; for example, comorbid MASLD and ALD have been termed MetALD while the less frequent combinational causes of CLD have not been formally named.^90^ Given these changes, many more patients with CLD will be considered having a component of MASLD, and potentially benefit from aggressive lifestyle modification.

While the contribution of obesity to the development of CLD is clear, there is limited evidence on the impact of diet and exercise-mediated weight loss on CLD-related outcomes.^31^ One challenge is parsing out the respective impacts of exercise, diet, and weight loss, as often trials employ multimodal approaches to optimization of weight and metabolic health. In terms of dietary strategies, there is reason to believe that certain interventions may help to improve MASLD as they can help with insulin resistance in the metabolic syndrome. Dietary interventions of interest include intermittent fasting (IF), low-carbohydrate diet, and the Mediterranean diet (MD).

IF is a dietary strategy to optimize metabolic health; thus, there is some research that indicates that it may help to reduce hepatic fat in those with MASLD/MASH. One systematic review and meta-analysis found that over a short-term period of strict IF there was a significant decrease in body weight, BMI, waist-hip ratio, total triglycerides and low density lipoprotein.^91^ Furthermore, the investigators found decreased liver enzyme levels, intrahepatic lipid content measured by magnetic resonance imaging, and liver stiffness measured by elastography. This study indicates that there may be promising benefits for the use of IF for the amelioration of MASLD.^91^

The efficacy of the low-carbohydrate diet has also been explored in MASLD. The underlying idea is that carbohydrate restriction can reduce body weight and reduce insulin resistance associated with metabolic syndrome.^92^, ^93^, ^94^ A randomized controlled trial evaluated the effect of low-carb high-fat (LCHF) diet on MASLD by comparing its effect on hepatic steatosis to IF and standard of care, which involved general dietary advice. The trial found a significant reduction in liver fat within all 3 groups but a significantly greater reduction in the LCHF and IF cohorts. There was no significant difference in hepatic steatosis reduction between LCHF and IF. Notably, liver stiffness significantly decreased in the IF group only. After 12 weeks, the trial supported that LCHF and IF both showed benefit in reducing steatosis in MASLD.^95^

The MD, known for its cardiometabolic benefits, is another intervention which may be helpful in reducing hepatic fat in those with MASLD. This dietary pattern is a primarily plant-based diet high in fiber, fruits, vegetables and legumes and low in dairy and animal protein consisting of higher levels of monounsaturated fats and omega 3 fatty acids. Current studies are limited to a few interventional and observational studies. In one case–control study, the MD and lifestyle changes cohort was associated with lower likelihood of hepatic steatosis, independent of both body weight and energy intake.^96^ A prospective study performed in Spain showed that a ketogenic MD prescribed to 14 obese men already with MASLD and metabolic syndrome significantly reduced hepatic steatosis and liver transaminase levels.^96^

## Complications

### HE

Specific considerations in patients with HE includes frailty, sarcopenia, and BCAA supplementation. HE is associated with higher rates of frailty (odds ratio 2.45) in patients with LC on transplant lists.^86^ Furthermore, in a cohort of patients hospitalized for LC, muscle depletion was noted to be associated with higher rates of MHE and HE (49% vs 30% and 30% vs 15%, respectively).^4^ While BCAA supplementation has not been shown to reduce mortality, QoL or nutritional parameters, a Cochrane systematic review found that oral formulations improved HE (notably intravenous formulations did not) in both MHE and OHE.^97^ A 2023 randomized control trial comparing intravenous BCAA supplementation with lactulose vs lactulose alone found short-term benefits of BCAA such as improvement in shorter intensive care unit stay and encephalopathy score, the latter of which was not sustained at day 3 or 7.^98^ Further head-to-head trials are warranted to investigate the impact of oral BCAA supplementation in HE. Additionally, further trials comparing them alongside and against nonabsorbable disaccharides or antibiotics are needed.^99^^,^^100^ Lastly, further work is needed to identify optimal dosing of BCAA, as prior studies have used doses ranging from 5.5 to 30 g/day. Given the uncertainty in the literature, BCAA supplementation for prevention of HE recurrence is currently not recommended in the outpatient setting.

Zinc is a cofactor for 2 enzymes for urea cycle as well as glutamine synthetase facilitating ammonia clearance in hepatocyte and skeletal muscle (PMID 25940731). Its deficiency has been implicated in CLD and HE. In CLD, low serum levels of zinc have been found in 83%–94% adult patients with lower levels corresponding with higher Child-Pugh scores and HE.^101^^,^^102^ Data on the impact of zinc supplementation on mental performance have been conflicting. One randomized control trial comparing oral zinc supplementation with lactulose compared to lactulose alone in patients with cirrhosis and HE demonstrated nonsignificant improvements in psychometric testing.^103^ Another study by Takuma et al from 2010 showed zinc supplementation significantly decreased HE grade, blood ammonia levels, neuro tests compared to standard therapy.^104^ Given conflicting evidence and need for more robust studies, currently only patients with confirmed zinc deficiency should be supplemented in accordance with general recommendations for zinc.

### Ascites

Specific dietary considerations for patients with ascites should include frailty, sodium management and transjugular intrahepatic porto-systemic shunt (TIPS). Ascites is associated with higher rates of frailty in patients with LC on transplant lists (adjusted OR (aOR) 1.56).^86^ Further work is needed to verify if higher rates of frailty are also seen in patients with LC not on transplant lists, though it can reasonably be extrapolated to these populations. In patients that have evidence of ascites, careful management of sodium intake is universally accepted among clinical practice guidelines to limit reaccumulation, discomfort and serial paracentesis. Guidelines differ for specific sodium targets, but general recommendations range from 4.6 to 6.9 grams of salt daily.^105^ However, evidence of salt restrictions’ efficacy in limiting accumulation and potential harms in the setting of frequent concomitance with hyponatremia warrant further investigation.^106^^,^^107^ Liberalization of diet can be considered in patients who are experiencing harm from a salt restriction—particularly those who are struggling to maintain their nutrition on a salt-restricted diet given impaired food taste.

In addition to considerations of sodium management, there is recent strong evidence for medically tailored meals (MTMs) from the SALTYFOOD trial for management of ascites. In this study, 40 patients who had an extensive history of multiple paracentesis were randomized to MTM (daily intake: <2000 mg sodium, >2100 kcal, >80 g of protein and nocturnal protein supplementation) and standard of care which involved providing the patient with an education handout about sodium restriction. Both interventions were able to reduce the number of therapeutic paracenteses; however, MTM was associated with reduced mortality, hospital length of stay, and modifications to diuretic strategy throughout the clinical course.^108^

Beyond management of serial paracenteses and sodium, TIPS has been shown to improve nutritional status in people with LC. TIPS is a procedure that has been shown to reduce portal hypertension, decreasing the severity and frequency of refractory ascites, as well as reducing variceal bleeding.^109^ However, nutritional status is an area of concern before and after TIPS placement.

It is known that malnutrition and sarcopenia adversely affect survival rate in patients with decompensated LC. Severe sarcopenia prior to TIPS placement can indicate worse outcomes, thus prompting the consensus that sarcopenia itself should not be the indicator driving the decision for TIPS placement.^110^ However, there are many studies which show improvement in overall nutritional status after the TIPS procedure.

Several studies have shown improvement in body composition after TIPS procedure, such as a beneficial increase to BMI or weight.^110^, ^111^, ^112^ Some research has found improvement in adipose tissue index in patients after TIPS while having a decrease in visceral adipose tissue, which was found to be associated with a lower rate of post-TIPS HE.^113^, ^114^, ^115^ Along with this beneficial increase in adipose tissue, there is evidence that shows improvement in skeletal muscle mass after TIPS. Notably, patients who were more sarcopenic and had a worse nutritional status preprocedure were found to have the most increase in skeletal muscle, adipose tissue and overall improvement in nutritional status.^109^^,^^112^^,^^113^^,^^116^ One study has even refuted that baseline sarcopenia did not significantly increase mortality risk or HE in TIPS patients. They found that TIPS may have a prognostic benefit in patients with baseline sarcopenia by improving malnourishment.^117^ Another important finding was that TIPS helped to improve the quality of muscle as a measure of myosteatosis.^118^

Although it should be determined individually, the evidence supports the role of TIPS in sarcopenic patients with refractory ascites requiring paracentesis. As a result of these studies, TIPS as an intervention for appropriate candidates with refractory ascites and variceal bleeding may also have the benefit of improving nutritional status postprocedure.

### The Role of the Registered Dietitian (RD)

The registered dietitian (RD) along with the interdisciplinary care team play an important role in managing the patient with CLD, whether the condition is acute or chronic. The RD conducts a comprehensive nutrition assessment which includes a diet history, a nutrition-focused physical exam (to assess lean body mass, adipose, signs and symptoms of micronutrient deficiencies) as well as anthropometric assessments to assess adipose stores and determine presence of sarcopenia. Additionally, the RD provides education and counseling on a wide range of topics, depending on the needs of the patient and the patients’ medical diagnosis to optimize intake of protein, calories, fluids and vitamins/minerals intake.

In critically ill patients with, the RD assesses specific energy, protein and fluid needs and makes recommendations for either enteral or parenteral nutrition support. In the case of enteral nutrition support, the RD makes recommendations for the type of enteral nutrition formula, the infusion rate and monitors for gastrointestinal tolerance. If parenteral nutrition support is warranted, the RD makes recommendations for the specific amounts of carbohydrates, amino acids, and lipids while monitoring labs (blood glucose, electrolytes), weight, and fluid status.

## Conclusion

Malnutrition, sarcopenia and frailty are highly prevalent among patients with CLD and warrant individualized approaches to nutritional optimization. This review describes several assessment tools to evaluate for malnutrition and sarcopenia including BMI, CPG, CT scanning, DEXA scanning, body measurements as well as screening surveys such as RFH-NPT and LDUST. It also describes known pathophysiologic mechanisms driving malnutrition and summarizes evidence-based recommendations from EASL and American Association for the Study of Liver Diseases regarding caloric, protein, sodium, and vitamin supplementation in patients with CLD. Lastly, we outline specific approaches for optimization of nutrition for patients with CLD based on etiology of disease and known complications.

## References

[1] Shin S., Jun D.W., Saeed W.K. (2021). A narrative review of malnutrition in chronic liver disease. Ann Transl Med.

[2] Dasarathy S. (2012). Consilience in sarcopenia of cirrhosis. J Cachexia Sarcopenia Muscle.

[3] Merli M., Lucidi C., Giannelli V. (2010). Cirrhotic patients are at risk for health care-associated bacterial infections. Clin Gastroenterol Hepatol.

[4] Merli M., Giusto M., Lucidi C. (2013). Muscle depletion increases the risk of overt and minimal hepatic encephalopathy: results of a prospective study. Metab Brain Dis.

[5] Huisman E.J., Trip E.J., Siersema P.D. (2011). Protein energy malnutrition predicts complications in liver cirrhosis. Eur J Gastroenterol Hepatol.

[6] European Association for the Study of the Liver (2019). EASL Clinical Practice Guidelines on nutrition in chronic liver disease. J Hepatol.

[7] Englesbe M.J., Patel S.P., He K. (2010). Sarcopenia and mortality after liver transplantation. J Am Coll Surg.

[8] Soeters P.B., Schols A.M. (2009). Advances in understanding and assessing malnutrition. Curr Opin Clin Nutr Metab Care.

[9] Carey E.J., Lai J.C., Wang C.W. (2017). A multicenter study to define sarcopenia in patients with end-stage liver disease. Liver Transpl.

[10] Lai J.C., Tandon P., Bernal W. (2021). Malnutrition, frailty, and sarcopenia in patients with cirrhosis: 2021 practice guidance by the American association for the study of liver diseases. Hepatology.

[11] Tandon P., Ney M., Irwin I. (2012). Severe muscle depletion in patients on the liver transplant wait list: its prevalence and independent prognostic value. Liver Transpl.

[12] Haj Ali S., Abu Sneineh A., Hasweh R. (2022). Nutritional assessment in patients with liver cirrhosis. World J Hepatol.

[13] Park J.H., Kang M., Jun D.W. (2021). Determining whether low protein intake (<1.0 g/kg) is a risk factor for malnutrition in patients with cirrhosis. J Clin Med.

[14] Mazeaud S., Zupo R., Couret A. (2023). Prevalence of sarcopenia in liver cirrhosis: a systematic review and meta-analysis. Clin Transl Gastroenterol.

[15] Duerksen D.R., Laporte M., Jeejeebhoy K. (2021). Evaluation of nutrition status using the subjective global assessment: malnutrition, cachexia, and sarcopenia. Nutr Clin Pract.

[16] Hanai T., Shiraki M., Nishimura K. (2021). Nutritional assessment tool for predicting sarcopenia in chronic liver disease. JCSM Rapid Commun.

[17] Moctezuma-Velazquez C., Ebadi M., Bhanji R.A. (2019). Limited performance of subjective global assessment compared to computed tomography-determined sarcopenia in predicting adverse clinical outcomes in patients with cirrhosis. Clin Nutr.

[18] Amodio P., Bemeur C., Butterworth R. (2013). The nutritional management of hepatic encephalopathy in patients with cirrhosis: international society for hepatic encephalopathy and nitrogen metabolism consensus. Hepatology.

[19] Borhofen S.M., Gerner C., Lehmann J. (2016). The royal free hospital-nutritional prioritizing tool is an independent predictor of deterioration of liver function and survival in cirrhosis. Dig Dis Sci.

[20] McFarlane M., Hammond C., Roper T. (2018). Comparing assessment tools for detecting undernutrition in patients with liver cirrhosis. Clin Nutr ESPEN.

[21] Casas-Deza D., Bernal-Monterde V., Betoré-Glaria E. (2023). Liver disease undernutrition screening tool questionnaire predicts decompensation and mortality in cirrhotic outpatients with portal hypertension. Nutrients.

[22] Georgiou A., Papatheodoridis G.V., Alexopoulou A. (2019). Evaluation of the effectiveness of eight screening tools in detecting risk of malnutrition in cirrhotic patients: the kirrhos study. Br J Nutr.

[23] Booi A.N., Menendez J., Norton H.J. (2015). Validation of a screening tool to identify undernutrition in ambulatory patients with liver cirrhosis. Nutr Clin Pract.

[24] Tandon P., Low G., Mourtzakis M. (2016). A model to identify sarcopenia in patients with cirrhosis. Clin Gastroenterol Hepatol.

[25] Alberino F., Gatta A., Amodio P. (2001). Nutrition and survival in patients with liver cirrhosis. Nutrition.

[26] Teiusanu A., Andrei M., Arbanas T. (2012). Nutritional status in cirrhotic patients. Maedica (Bucur).

[27] Cichoż-Lach H., Michalak A. (2017). A comprehensive review of bioelectrical impedance analysis and other methods in the assessment of nutritional status in patients with liver cirrhosis. Gastroenterol Res Pract.

[28] Iwaki M., Kobayashi T., Nogami A. (2023). Impact of sarcopenia on non-alcoholic fatty liver disease. Nutrients.

[29] Glass C., Hipskind P., Tsien C. (2013). Sarcopenia and a physiologically low respiratory quotient in patients with cirrhosis: a prospective controlled study. J Appl Physiol (1985).

[30] Glass C., Hipskind P., Cole D. (2012). Handheld calorimeter is a valid instrument to quantify resting energy expenditure in hospitalized cirrhotic patients: a prospective study. Nutr Clin Pract.

[31] (2019). Easl clinical practice guidelines on nutrition in chronic liver disease. J Hepatol.

[32] Dasarathy S., Merli M. (2016). Sarcopenia from mechanism to diagnosis and treatment in liver disease. J Hepatol.

[33] Guglielmi F.W., Panella C., Buda A. (2005). Nutritional state and energy balance in cirrhotic patients with or without hypermetabolism. Multicentre prospective study by the ‘nutritional problems in gastroenterology’ section of the Italian society of gastroenterology (sige). Dig Liver Dis.

[34] Riggio O., Angeloni S., Ciuffa L. (2003). Malnutrition is not related to alterations in energy balance in patients with stable liver cirrhosis. Clin Nutr.

[35] Nielsen K., Kondrup J., Martinsen L. (1995). Long-term oral refeeding of patients with cirrhosis of the liver. Br J Nutr.

[36] Prieto-Frías C., Conchillo M., Payeras M. (2016). Factors related to increased resting energy expenditure in men with liver cirrhosis. Eur J Gastroenterol Hepatol.

[37] Peng S., Plank L.D., McCall J.L. (2007). Body composition, muscle function, and energy expenditure in patients with liver cirrhosis: a comprehensive study. Am J Clin Nutr.

[38] Greco A.V., Mingrone G., Benedetti G. (1998). Daily energy and substrate metabolism in patients with cirrhosis. Hepatology.

[39] Roubenoff R., Roubenoff R.A., Cannon J.G. (1994). Rheumatoid cachexia: cytokine-driven hypermetabolism accompanying reduced body cell mass in chronic inflammation. J Clin Invest.

[40] Lin S.Y., Wang Y.Y., Sheu W.H. (2002). Increased serum leptin concentrations correlate with soluble tumour necrosis factor receptor levels in patients with cirrhosis. Clin Endocrinol.

[41] Shinsyu A., Bamba S., Kurihara M. (2020). Inflammatory cytokines, appetite-regulating hormones, and energy metabolism in patients with gastrointestinal cancer. Oncol Lett.

[42] Paulsen Ø., Laird B., Aass N. (2017). The relationship between pro-inflammatory cytokines and pain, appetite and fatigue in patients with advanced cancer. PLoS One.

[43] Bajaj J.S., Kakiyama G., Zhao D. (2017). Continued alcohol misuse in human cirrhosis is associated with an impaired gut-liver axis. Alcohol Clin Exp Res.

[44] Chen P., Stärkel P., Turner J.R. (2015). Dysbiosis-induced intestinal inflammation activates tumor necrosis factor receptor i and mediates alcoholic liver disease in mice. Hepatology.

[45] Horvath A., Rainer F., Bashir M. (2019). Biomarkers for oralization during long-term proton pump inhibitor therapy predict survival in cirrhosis. Sci Rep.

[46] Davcev P., Vanovski B., Sestakov D. (1969). Protein-losing enteropathy in patients with liver cirrhosis. Digestion.

[47] Dahlqvist G.E., Jamar F., Zech F. (2012). In-111 transferrin scintigraphy in cirrhosis with hypoalbuminemia: evidence for protein-losing enteropathy in a small group of selected cases. Scand J Gastroenterol.

[48] Takada A., Kobayashi K., Takeuchi J. (1970). Gastroenteric clearance of albumin in liver icrrhosis; relative protein-losing gastroenteropathy. Digestion.

[49] Kinny-Köster B., Bartels M., Becker S. (2016). Plasma amino acid concentrations predict mortality in patients with end-stage liver disease. PLoS One.

[50] McCullough A.J., Mullen K.D., Kalhan S.C. (1992). Body cell mass and leucine metabolism in cirrhosis. Gastroenterology.

[51] Morgan M.Y., Marshall A.W., Milsom J.P. (1982). Plasma amino-acid patterns in liver disease. Gut.

[52] Record C.O., Buxton B., Chase R.A. (1976). Plasma and brain amino acids in fulminant hepatic failure and their relationship to hepatic encephalopathy. Eur J Clin Invest.

[53] Grossmann M., Hoermann R., Gani L. (2012). Low testosterone levels as an independent predictor of mortality in men with chronic liver disease. Clin Endocrinol.

[54] Griggs R.C., Kingston W., Jozefowicz R.F. (1989). Effect of testosterone on muscle mass and muscle protein synthesis. J Appl Physiol (1985).

[55] Alves B.C., Luchi-Cruz M.M., Lopes A.B. (2023). Predicting dry weight in patients with cirrhotic ascites undergoing large-volume paracentesis. Clin Nutr ESPEN.

[56] Bischoff S.C., Bernal W., Dasarathy S. (2020). Espen practical guideline: clinical nutrition in liver disease. Clin Nutr.

[57] Soulsby C.T., Morgan M.Y. (1999). Dietary management of hepatic encephalopathy in cirrhotic patients: survey of current practice in United Kingdom. BMJ.

[58] Summerskill W.H., Wolfe S.J., Davidson C.S. (1957). The management of hepatic coma in relation to protein withdrawal and certain specific measures. Am J Med.

[59] Anand A.C. (2017). Nutrition and muscle in cirrhosis. J Clin Exp Hepatol.

[60] Córdoba J., López-Hellín J., Planas M. (2004). Normal protein diet for episodic hepatic encephalopathy: results of a randomized study. J Hepatol.

[61] Shaw J., Tate V., Hanson J. (2020). What diet should i recommend my patient with hepatic encephalopathy?. Curr Hepatol Rep.

[62] Li G., Li Z., Liu J. (2024). Amino acids regulating skeletal muscle metabolism: mechanisms of action, physical training dosage recommendations and adverse effects. Nutr Metab (Lond).

[63] Gluud L.L., Dam G., Borre M. (2013). Oral branched-chain amino acids have a beneficial effect on manifestations of hepatic encephalopathy in a systematic review with meta-analyses of randomized controlled trials. J Nutr.

[64] Bianchi G.P., Marchesini G., Fabbri A. (1993). Vegetable versus animal protein diet in cirrhotic patients with chronic encephalopathy. A randomized cross-over comparison. J Intern Med.

[65] Uribe M., Márquez M.A., Garcia Ramos G. (1982). Treatment of chronic portal--systemic encephalopathy with vegetable and animal protein diets. A controlled crossover study. Dig Dis Sci.

[66] Venu M., Martin E., Saeian K. (2013). High prevalence of vitamin a deficiency and vitamin d deficiency in patients evaluated for liver transplantation. Liver Transpl.

[67] Petta S., Cammà C., Scazzone C. (2010). Low vitamin d serum level is related to severe fibrosis and low responsiveness to interferon-based therapy in genotype 1 chronic hepatitis c. Hepatology.

[68] Ng V.L. (2009). Liver disease, coagulation testing, and hemostasis. Clin Lab Med.

[69] Agarwal B., Wright G., Gatt A. (2012). Evaluation of coagulation abnormalities in acute liver failure. J Hepatol.

[70] Jin S., Hong L., FakhriRavari A. (2022). The role of vitamin K in cirrhosis: do pharmaco-k-netics matter?. Gastrointest Disord.

[71] Zarghamravanbakhsh P., Frenkel M., Poretsky L. (2021). Metabolic causes and consequences of nonalcoholic fatty liver disease (NAFLD). Metabol Open.

[72] Marjot T., Tomlinson J.W., Hodson L. (2023). Timing of energy intake and the therapeutic potential of intermittent fasting and time-restricted eating in NAFLD. Gut.

[73] Plank L.D., Gane E.J., Peng S. (2008). Nocturnal nutritional supplementation improves total body protein status of patients with liver cirrhosis: a randomized 12-month trial. Hepatology.

[74] Hanai T., Shiraki M., Imai K. (2020). Late evening snack with branched-chain amino acids supplementation improves survival in patients with cirrhosis. J Clin Med.

[75] Chen C.J., Wang L.C., Kuo H.T. (2019). Significant effects of late evening snack on liver functions in patients with liver cirrhosis: a meta-analysis of randomized controlled trials. J Gastroenterol Hepatol.

[76] DiMartini A., Cruz R.J., Dew M.A. (2013). Muscle mass predicts outcomes following liver transplantation. Liver Transpl.

[77] Dasarathy J., McCullough A.J., Dasarathy S. (2017). Sarcopenia in alcoholic liver disease: clinical and molecular advances. Alcohol Clin Exp Res.

[78] Estruch R., Sacanella E., Fernández-Solá J. (1998). Natural history of alcoholic myopathy: a 5-year study. Alcohol Clin Exp Res.

[79] Bhanji R.A., Narayanan P., Moynagh M.R. (2019). Differing impact of sarcopenia and frailty in nonalcoholic steatohepatitis and alcoholic liver disease. Liver Transpl.

[80] Lai J.C., Dodge J.L., Kappus M.R. (2020). Changes in frailty are associated with waitlist mortality in patients with cirrhosis. J Hepatol.

[81] Carias S., Castellanos A.L., Vilchez V. (2016). Nonalcoholic steatohepatitis is strongly associated with sarcopenic obesity in patients with cirrhosis undergoing liver transplant evaluation. J Gastroenterol Hepatol.

[82] Hiraoka A., Michitaka K., Ueki H. (2016). Sarcopenia and two types of presarcopenia in Japanese patients with chronic liver disease. Eur J Gastroenterol Hepatol.

[83] Bering T., Diniz K.G.D., Coelho M.P.P. (2018). Association between pre-sarcopenia, sarcopenia, and bone mineral density in patients with chronic hepatitis C. J Cachexia Sarcopenia Muscle.

[84] Tantai X., Liu Y., Yeo Y.H. (2022). Effect of sarcopenia on survival in patients with cirrhosis: a meta-analysis. J Hepatol.

[85] Tuo S., Yeo Y.H., Chang R. (2024). Prevalence of and associated factors for sarcopenia in patients with liver cirrhosis: a systematic review and meta-analysis. Clin Nutr.

[86] Lai J.C., Rahimi R.S., Verna E.C. (2019). Frailty associated with waitlist mortality independent of ascites and hepatic encephalopathy in a multicenter study. Gastroenterology.

[87] Nakamura A., Yoshimura T., Ichikawa T. (2023). Liver disease-related sarcopenia: a predictor of poor prognosis by accelerating hepatic decompensation in advanced chronic liver disease. Cureus.

[88] Kim G., Kang S.H., Kim M.Y. (2017). Prognostic value of sarcopenia in patients with liver cirrhosis: a systematic review and meta-analysis. PLoS One.

[89] Skladany L., Molcan P., Vnencakova J. (2021). Frailty in nonalcoholic fatty liver cirrhosis: a comparison with alcoholic cirrhosis, risk patterns, and impact on prognosis. Can J Gastroenterol Hepatol.

[90] Rinella M.E., Lazarus J.V., Ratziu V. (2023). A multisociety delphi consensus statement on new fatty liver disease nomenclature. Hepatology.

[91] Lange M., Nadkarni D., Martin L. (2023). Intermittent fasting improves hepatic end points in nonalcoholic fatty liver disease: a systematic review and meta-analysis. Hepatol Commun.

[92] Feinman R.D., Pogozelski W.K., Astrup A. (2015). Dietary carbohydrate restriction as the first approach in diabetes management: critical review and evidence base. Nutrition.

[93] Volek J.S., Phinney S.D., Forsythe C.E. (2009). Carbohydrate restriction has a more favorable impact on the metabolic syndrome than a low fat diet. Lipids.

[94] Skytte M.J., Samkani A., Petersen A.D. (2019). A carbohydrate-reduced high-protein diet improves HbA(1c) and liver fat content in weight stable participants with type 2 diabetes: a randomised controlled trial. Diabetologia.

[95] Holmer M., Lindqvist C., Petersson S. (2021). Treatment of NAFLD with intermittent calorie restriction or low-carb high-fat diet - a randomised controlled trial. JHEP Rep.

[96] Katsagoni C.N., Georgoulis M., Papatheodoridis G.V. (2017). Associations between lifestyle characteristics and the presence of nonalcoholic fatty liver disease: a case-control study. Metab Syndr Relat Disord.

[97] Gluud L.L., Dam G., Les I. (2017). Branched-chain amino acids for people with hepatic encephalopathy. Cochrane Database Syst Rev.

[98] Mehtani R., Premkumar M., Garg S. (2023). Intravenous BCAA infusion does not lead to a sustained recovery from overt he in aclf - an open label randomized clinical trial. J Clin Exp Hepatol.

[99] Cabré E., Rodríguez-Iglesias P., Caballería J. (2000). Short- and long-term outcome of severe alcohol-induced hepatitis treated with steroids or enteral nutrition: a multicenter randomized trial. Hepatology.

[100] Baltz J.G., Argo C.K., Al-Osaimi A.M. (2010). Mortality after percutaneous endoscopic gastrostomy in patients with cirrhosis: a case series. Gastrointest Endosc.

[101] Teriaky A., Mosli M., Chandok N. (2017). Prevalence of fat-soluble vitamin (a, d, and e) and zinc deficiency in patients with cirrhosis being assessed for liver transplantation. Acta Gastroenterol Belg.

[102] Sengupta S., Wroblewski K., Aronsohn A. (2015). Screening for zinc deficiency in patients with cirrhosis: when should we start?. Dig Dis Sci.

[103] Bresci G., Parisi G., Banti S. (1993). Management of hepatic encephalopathy with oral zinc supplementation: a long-term treatment. Eur J Med.

[104] Takuma Y., Nouso K., Makino Y. (2010). Clinical trial: oral zinc in hepatic encephalopathy. Aliment Pharmacol Ther.

[105] Theodoridis X., Grammatikopoulou M.G., Petalidou A. (2020). A systematic review of medical nutrition therapy guidelines for liver cirrhosis: do we agree?. Nutr Clin Pract.

[106] Gu X.B., Yang X.J., Zhu H.Y. (2012). Effect of a diet with unrestricted sodium on ascites in patients with hepatic cirrhosis. Gut Liver.

[107] Sorrentino P., Castaldo G., Tarantino L. (2012). Preservation of nutritional-status in patients with refractory ascites due to hepatic cirrhosis who are undergoing repeated paracentesis. J Gastroenterol Hepatol.

[108] Tapper E.B., Baki J., Nikirk S. (2020). Medically tailored meals for the management of symptomatic ascites: the saltyfood pilot randomized clinical trial. Gastroenterol Rep (Oxf).

[109] Gazda J., Di Cola S., Lapenna L. (2023). The impact of transjugular intrahepatic portosystemic shunt on nutrition in liver cirrhosis patients: a systematic review. Nutrients.

[110] de Franchis R., Bosch J., Garcia-Tsao G. (2022). Baveno VII - renewing consensus in portal hypertension. J Hepatol.

[111] Pang N., Zhao C., Li J. (2021). Body mass index changes after transjugular intrahepatic portosystemic shunt in individuals with cirrhosis. Nutrition.

[112] Liu J., Ma J., Yang C. (2022). Sarcopenia in patients with cirrhosis after transjugular intrahepatic portosystemic shunt placement. Radiology.

[113] Gioia S., Ridola L., Cristofaro L. (2021). The improvement in body composition including subcutaneous and visceral fat reduces ammonia and hepatic encephalopathy after transjugular intrahepatic portosystemic shunt. Liver Int.

[114] Artru F., Miquet X., Azahaf M. (2020). Consequences of tipss placement on the body composition of patients with cirrhosis and severe portal hypertension: a large retrospective ct-based surveillance. Aliment Pharmacol Ther.

[115] Wang C., Teng Y., Gao J. (2023). Low adipose tissue index as an indicator of hepatic encephalopathy in cirrhotic patients following transjugular intrahepatic portosystemic shunt. Abdom Radiol (NY).

[116] Tsien C., Shah S.N., McCullough A.J. (2013). Reversal of sarcopenia predicts survival after a transjugular intrahepatic portosystemic stent. Eur J Gastroenterol Hepatol.

[117] Xiong B., Yang C., Zhou C. (2023). Tips placement as the first-line therapy to prevent variceal rebleeding in patients with cirrhosis and sarcopenia. Eur J Radiol.

[118] Gioia S., Merli M., Nardelli S. (2019). The modification of quantity and quality of muscle mass improves the cognitive impairment after tips. Liver Int.

